# Supplementary material for: Radiosensitization of Glioblastoma by the K-ras Inhibitor RMC-6236
Source: bioRxiv. 2026 Jun 2:2026.05.29.728724. Preprint. [Version 1] doi: 10.64898/2026.05.29.728724 (PMC13252110; doi:10.64898/2026.05.29.728724)

bioRxiv preprint doi: https://doi.org/10.64898/2026.05.29.728724; this version posted June 2, 2026. The copyright holder for this preprint (which was not certified by peer review) is the author/funder. This article is a US Government work. It is not subject to copyright under 17 USC 105 and is also made available for use under a CC0 license.

Supplementary Figure S1-1. Effect of radiation on KRAS mRNA expression in GBM cells

Quantitative RT-PCR analysis of KRAS mRNA expression in human GBM cell lines (U251, LN-18, ACPK1, and OSU61) following exposure to 4 Gy radiation at the indicated time points.

Supplementary Figure S1-2. Effect of radiation on KRAS protein stability in GBM cells.

Cycloheximide (CHX) chase assays were performed to assess KRAS protein stability. The cells were pretreated with CHX (200 or 500 μmol/L) for 4 h, followed by exposure to 4 Gy radiation. KRAS protein levels were analyzed by immunoblotting at the indicated time points.

Supplementary Figure S2-1. Representative images of radiation-induced γH2AX foci following RMC-6236 treatment in GBM cells

Representative immunofluorescence images of γH2AX foci in U251, LN-18, ACPK1, and OSU61 cells after treatment with RMC-6236 (10 nmol/L) and/or 4 Gy radiation. The cells were analyzed 1 and 24 h after irradiation. Images show control cells and cells treated with RMC-6236 alone, radiation alone, or a combination of RMC-6236 and radiation treatment. Scale bar = 20 μm.

Supplementary Figure S2-2. Representative images of mitotic catastrophe following combined RMC-6236 and radiation treatment in GBM cells

Representative immunofluorescence images of mitotic catastrophe in U251, LN-18, ACPK1, and OSU61 cells after treatment with RMC-6236 (10 nmol/L) and/or 4 Gy radiation. The cells were stained for α-tubulin and DAPI and analyzed at the indicated time points after irradiation.

bioRxiv preprint doi: https://doi.org/10.64898/2026.05.29.728724; this version posted June 2, 2026. The copyright holder for this preprint (which was not certified by peer review) is the author/funder. This article is a US Government work. It is not subject to copyright under 17 USC 105 and is also made available for use under a CC0 license.

Arrowheads indicate cells undergoing mitotic catastrophe characterized by multinucleation or nuclear fragmentation. Scale bar = 20 µm.

Supplementary Figure S3. Effect of RMC-6236 on cell cycle distribution in GBM cells

The cell cycle distribution was analyzed in U251, LN-18, ACPK1, and OSU61 cells following treatment with RMC-6236 (10 nmol/L) and/or 4 Gy radiation. The percentage of cells in the G0/G1, S, and G2/M phases was determined by flow cytometry at the indicated time points.

Supply 1-1. The effect of radiation on K-ras mRNA expression in 4 GBM cell lines with wild-type K-ras

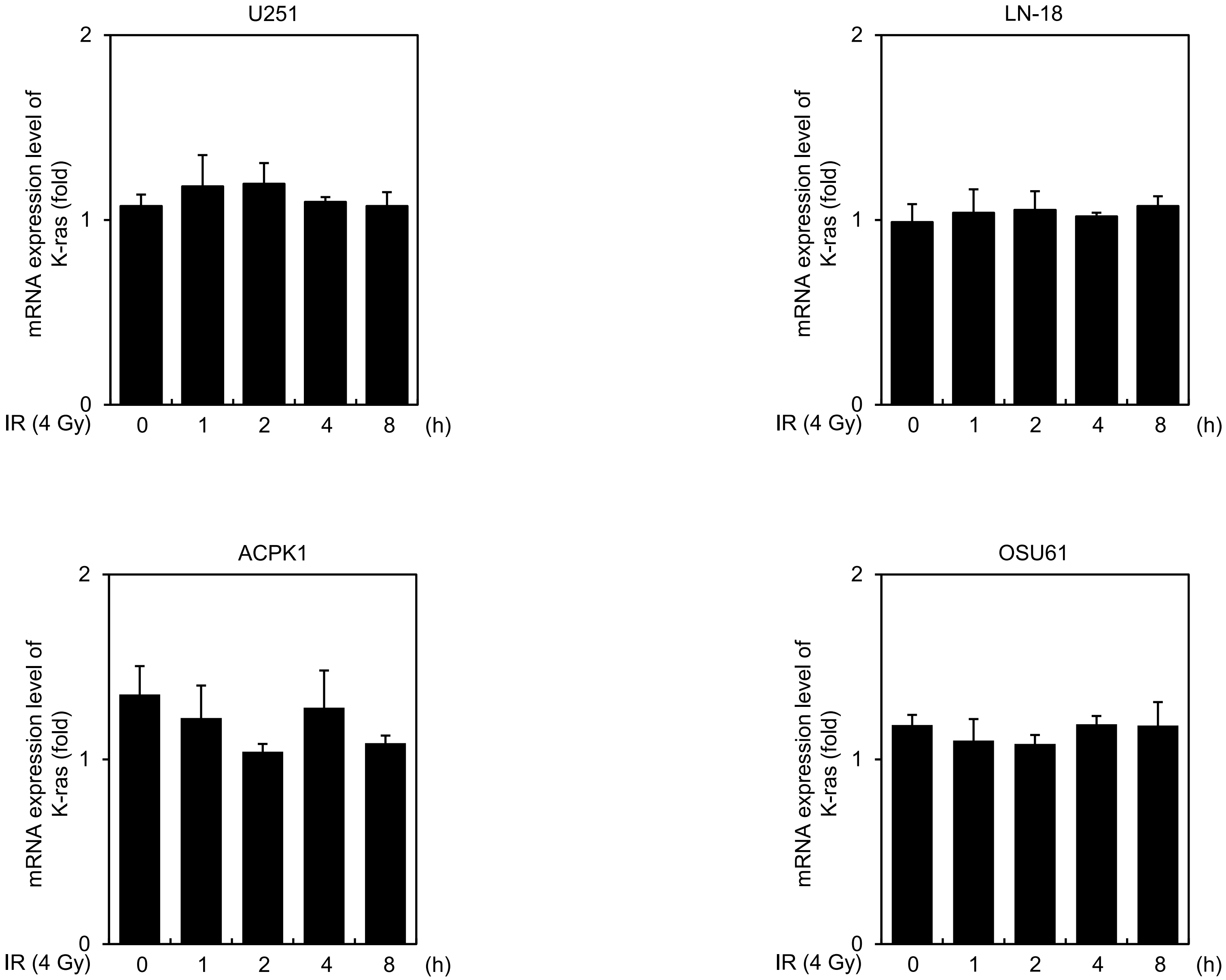

Supply 1-2. The treatment of CHX to 4 GBM cell lines

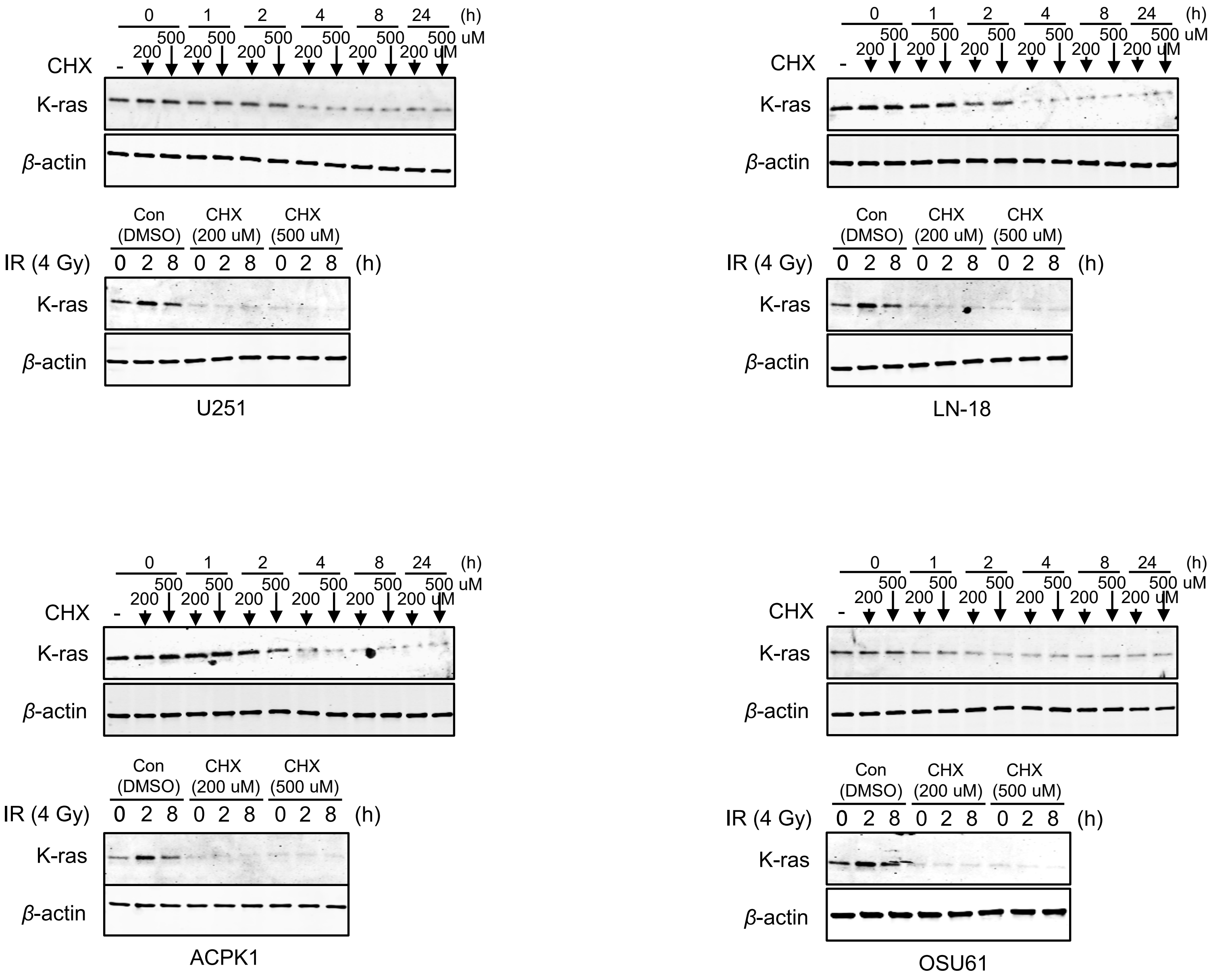

Supply 2_2. The histograph of radiation-induced Mitotic Catastrophe by RMC-6236 in GBM cell lines

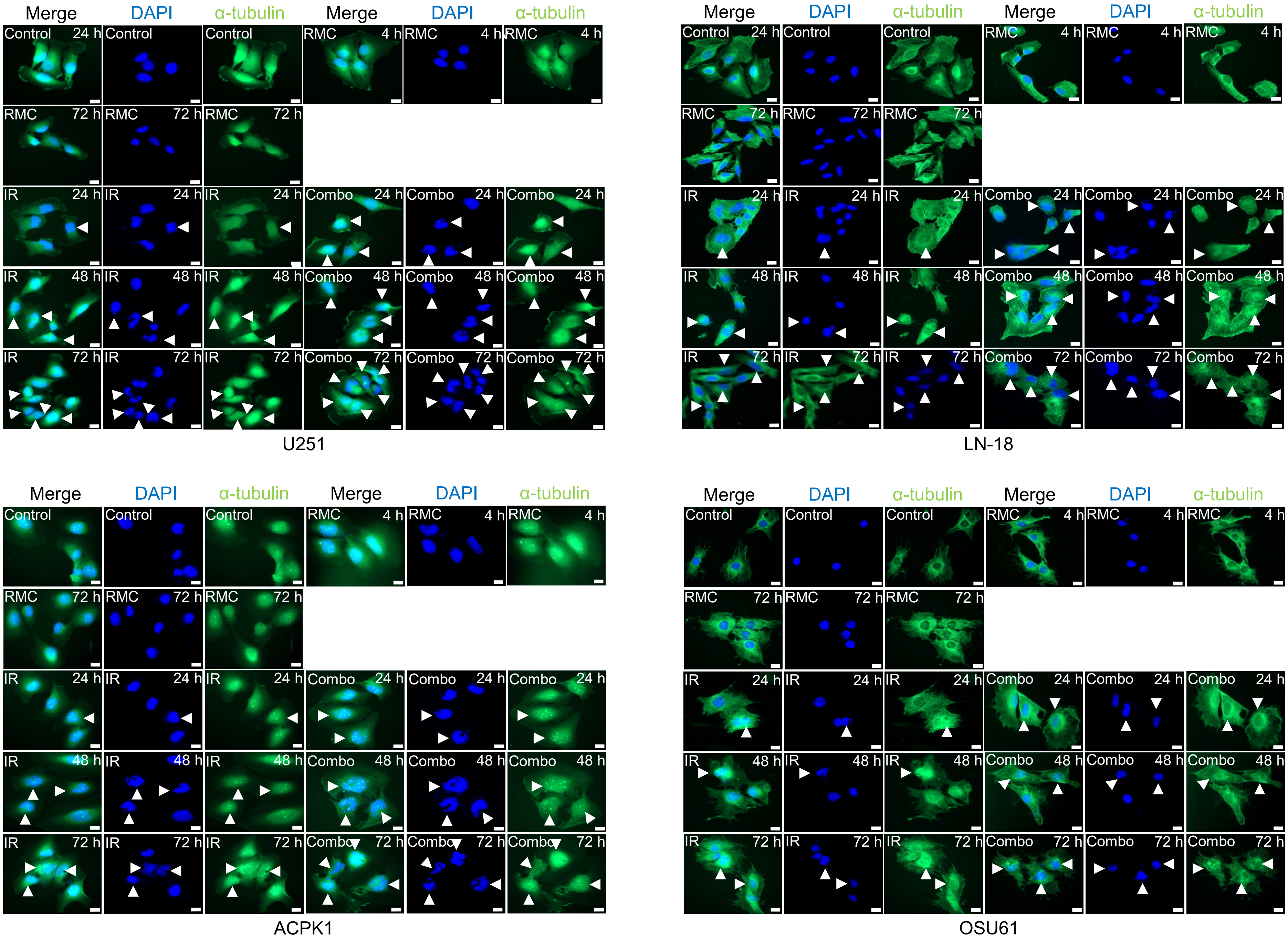

Supply 2_1. The histograph of radiation-induced **γ**-H2AX Foci by RMC-6236 in GBM cell lines

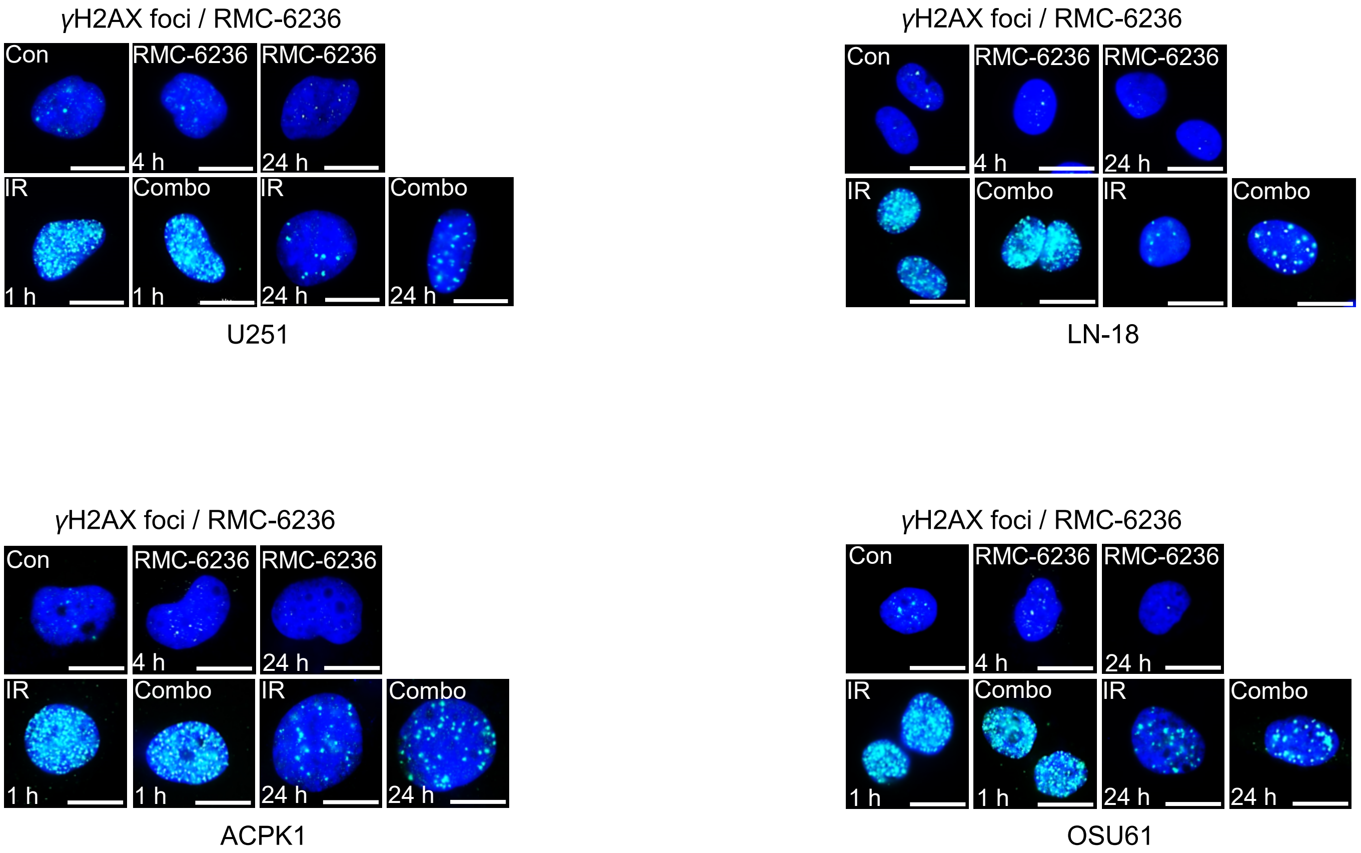

Supply 3. The effect of RMC-6236 on cell cycle in GBM cell lines

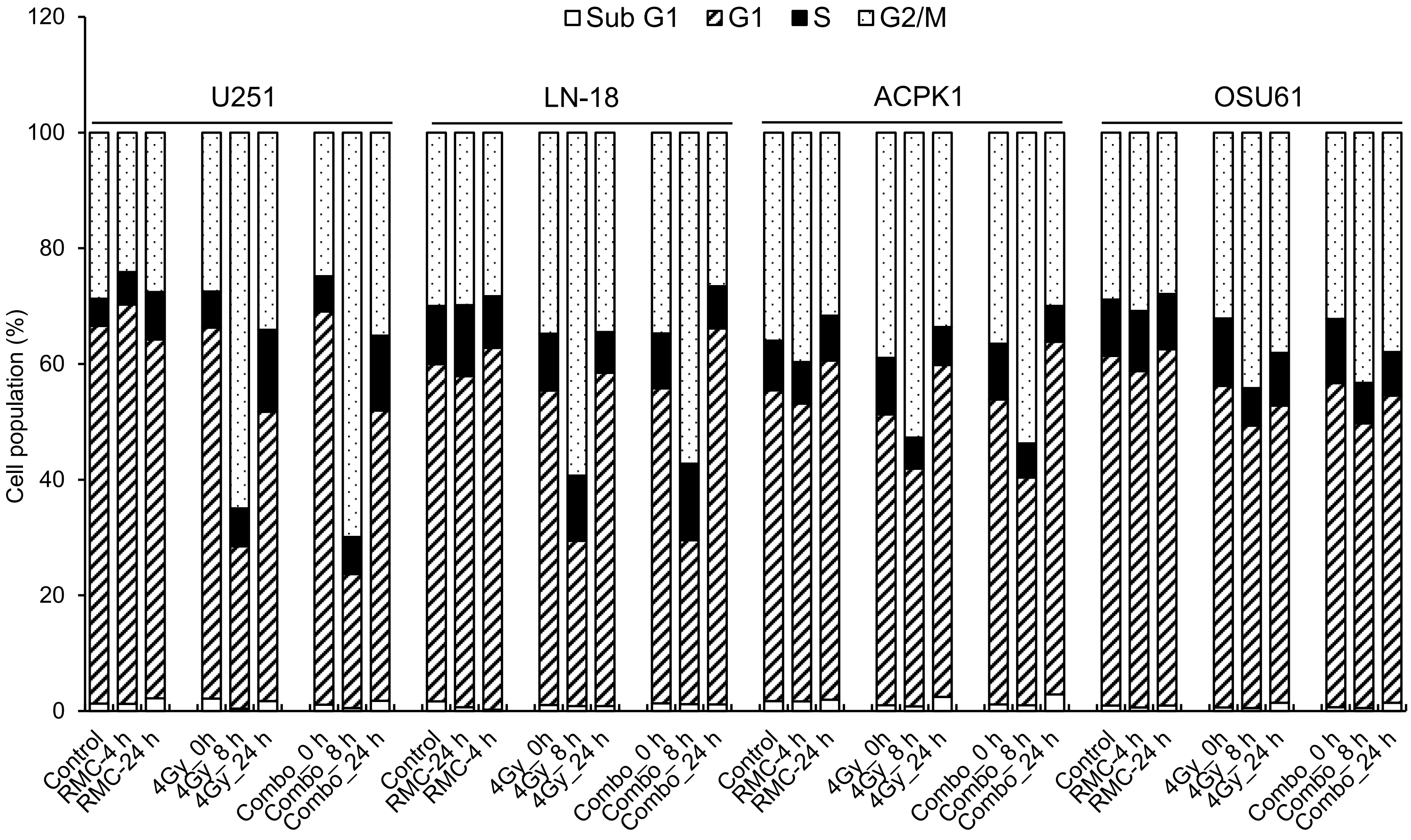

Supplement: Supplement 1 [file NIHPP2026.05.29.728724v1-supplement-1.pdf]
